# Development of Erythema Nodosum After Olaparib Treatment in a Patient With Recurrent Breast Cancer and BRCA2 Mutation: A Case Report

**DOI:** 10.7759/cureus.44864

**Published:** 2023-09-07

**Authors:** Masayuki Saito, Kimihito Fujii, Hirona Banno, Yukie Ito, Shogo Nakano

**Affiliations:** 1 Department of Surgery, Aichi Medical University, Nagakute, JPN

**Keywords:** skin rash, brca 1/2, breast cancer, erythema nodosum, olaparib

## Abstract

*BRCA1* and* 2* mutations are known to be associated with breast cancer, and olaparib, a poly (adenosine diphosphate-ribose) polymerase (PARP) inhibitor, has been shown to be effective in cells carrying these mutations in some studies. Erythema nodosum (EN), which is one adverse event of olaparib and is discussed in this paper, is considered to be a very rare condition.

A 69-year-old female patient underwent left breast conservative surgery with axillary lymph node dissection for left invasive ductal breast cancer (stage IIB). Her family history included a sister who developed ovarian cancer at age 63. Five years postoperatively, systemic metastases were discovered in the lung, bone, hilar, and poststernal lymph nodes. The surgically removed metastatic lung nodule was diagnosed as an estrogen receptor (ER)-positive, progesterone receptor (PgR)-negative, and human epidermal growth factor receptor 2 (HER2)-negative metastatic adenocarcinoma of breast cancer origin. And germline mutations of *BRCA1/*2 were assessed using BRACAnalysis CDx^®^ (Myriad Genetics, Salt Lake City, UT, USA), and *BRCA2* 1241 delC was identified as a deleterious mutation. Oral administration of olaparib was started. On day 4 of this treatment, numerous erythematous plaques characterized by intense tenderness and infiltration appeared on the extensor surfaces of the bilateral lower legs. On the basis of the clinical findings, the lesions were diagnosed as EN. Oral prednisolone was started at the same time as olaparib discontinuation, and the EN lesions disappeared in one week.

EN is an inflammatory lesion characterized by tender subcutaneous induration with a flushed surface, predominantly on the bilateral lower legs. EN occurring after olaparib administration is considered to be very rare. This article describes such a case and reviews the relevant literature.

## Introduction

*BRCA1* and *2* mutations are known to be associated with breast cancer [1], and olaparib, a poly (adenosine diphosphate-ribose) polymerase (PARP) inhibitor, has been shown to be effective in cells carrying these mutations in some studies [2-6]. The OlympiAD [2] and OlympiA [3] studies demonstrated the efficacy of olaparib in the maintenance treatment of unresectable or recurrent BRCA mutation-positive, human epidermal growth factor receptor 2 (HER2)-negative breast cancer previously treated with anticancer chemotherapy and in the adjuvant treatment of high-risk BRCA mutation-positive, HER2-negative breast cancer. In these clinical trials, the most common adverse events associated with olaparib were nausea, vomiting, fatigue, and anemia, with incidences of 58%, 29.8%, 28.8%, and 40% in the OlympiAD study and 56.9%, 22.6%, 40.1%, and 23.5% in the OlympiA study, respectively. Palmar-plantar erythrodysesthesia was the only skin-related adverse event in the OlympiAD study, with an incidence of 0.5%, while no cutaneous adverse events were reported in the OlympiA study [2,3]. Olaparib also showed clinical efficacy in ovarian cancer, prostate cancer, and pancreatic cancer [4-6]. In these studies, frequent adverse events were nausea, fatigue or asthenia, anemia, vomiting, and diarrhea. No skin disorders, including EN, were reported as adverse events [4-6].

EN is a form of septal panniculitis and an inflammatory lesion characterized by tender subcutaneous induration with a flushed surface, predominantly on the bilateral lower legs. Common causes of EN include drug reactions, strep throat, tuberculosis, sarcoidosis, Behcet’s disease, pregnancy, and inflammatory bowel disease [7].

Herein, we report a very rare case of erythema nodosum (EN) that developed after olaparib treatment of HER2-negative recurrent breast cancer with a *BRCA2* mutation.

## Case presentation

**Figure 1:**
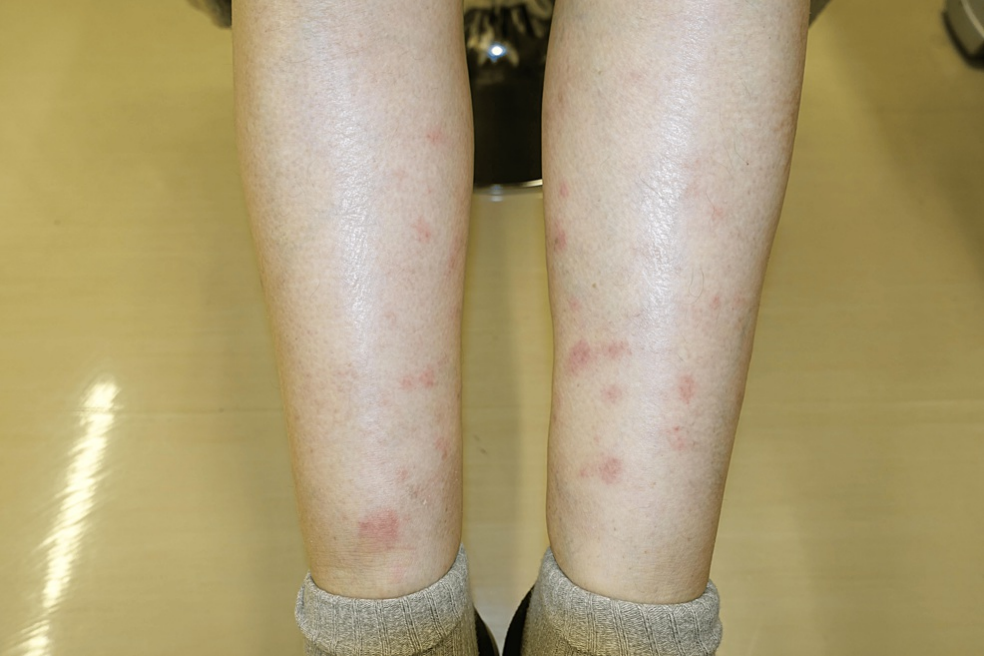
Skin rash on the bilateral lower legs. Numerous erythematous plaques with intense tenderness and infiltration on the extensor surfaces of the bilateral lower legs.

A 69-year-old female was diagnosed with left breast invasive ductal carcinoma (cT2N1M0, cStage IIB, estrogen receptor (ER) negative, progesterone receptor (PgR) negative, and HER2 negative). Her family medical history included a younger sister who developed ovarian cancer at age 63. After receiving neoadjuvant chemotherapy with 5-fluorouracil, epirubicin, and cyclophosphamide, followed by paclitaxel, she underwent left breast conservative surgery with axillary lymph node dissection. Radiotherapy for the breast remnant was performed postoperatively. Histopathological examination of the resected specimen showed a complete pathological response. Five years postoperatively, a computed tomography (CT) scan revealed nodular lesions in both lungs, and the patient was referred to our hospital for further treatment. Positron emission tomography-computed tomography (PET-CT) demonstrated multiple metastases in the lung field, hilar and poststernal lymph nodes, and bone. The metastatic lung nodule was removed by video-assisted thoracic surgery for a definitive diagnosis and was identified as an ER-positive, PgR-negative, and HER2-negative metastatic adenocarcinoma of breast cancer origin. Treatment with fulvestrant, palbociclib, and denosumab prevented further development of these lesions for about 11 months. However, the metastatic disease spread systemically to the paraaortic and supraclavicular lymph nodes. Germline mutations of *BRCA1*/*2* were assessed using BRACAnalysis CDx® (Myriad Genetics, Salt Lake City, UT, USA), and *BRCA2* 1241 delC was identified as a deleterious mutation. Oral administration of olaparib, 600 mg/day, was started. Other than olaparib, no other drugs were newly initiated. On day 4 of this treatment, numerous erythematous plaques characterized by intense tenderness and infiltration appeared on the extensor surfaces of the bilateral lower legs (Figure 1). The patient had difficulty walking due to the tenderness. She had no symptoms other than a skin rash on both lower legs, associated pain, and difficulty walking. Laboratory tests before the onset of EN and after its resolution yielded similar results (Table 1). No drug reaction was identified. On the basis of the clinical findings, the lesions were diagnosed as EN. A biopsy of the affected skin lesion could not be done due to the patient's refusal. Also, we did not perform PET-CT at the onset of EN. Oral prednisolone, 20 mg/day, was started at the same time as olaparib discontinuation, and the EN lesions gradually changed to yellowish-brown and disappeared in one week. The patient’s disease status was kept stable for two years by treatment with tegafur, gimerasil, and oterasil potassium, but thereafter liver metastases developed. Intravenous chemotherapy with eribulin was subsequently performed. There has been no relapse of the cutaneous symptoms of EN since the first episode that occurred during olaparib treatment.

**Table 1:** Laboratory test results before EN onset and after its resolution.

Blood testing	Before olaparib administration	EN onset	After EN resolution	Normal range
White blood cells (/µL)	3300	2400	8300	(5000–8000)
Neutrophils (%)	65.5	67.1	88.5	(49.2–58.5)
Lymphocytes (%)	17.7	17.7	6.9	(32.7–42.7)
Monocytes (%)	13.8	11.9	4.1	(3.8–5.5)
Eosinophils (%)	2.4	2.9	0.4	(0.0–4.5)
Basophils (%)	0.6	0.4	0.1	(0.0–1.9)
Hemoglobin (g/dL)	11.7	11.5	12.4	(11.4–14.8)
Platelets (/µL)	284,000	302,000	333,000	(180,000–350,000)
Total bilirubin (mg/dL)	0.51	0.59	0.41	(0.30–1.20)
BUN (mg/dL)	12.3	10.1	14.6	(8.0–22.0)
Cre (mg/dL)	0.46	0.54	0.53	(0.40–0.70)
AST (U/L)	24	23	20	(13–33)
ALT (U/L)	17	18	17	(6–27)
ALP (U/L)	232	235	233	(115–359)
LD (U/L)	183	191	161	(119–229)
γ–GT (U/L)	22	23	25	(10–47)
CK (U/L)	71	82	39	(45–163)

## Discussion

The *BRCA1*/*2* genes encode proteins involved in repairing DNA double-strand breaks by homologous recombination [8]. Cells with defective homologous recombination repair, such as those with BRCA mutations, are sensitive to PARP inhibition mediated by several mechanisms, including PARP trapping on DNA at single-strand break sites. These processes prevent the repair of single-strand breaks, which in replicating cells leads to the formation of double-strand breaks that cannot be accurately repaired in tumors with defective homologous recombination repair. As a result, PARP inhibitors cause the accumulation of DNA damage and tumor cell death [9].

In breast cancer, olaparib showed clinical efficacy in the OlympiAD [2] and OlympiA [3] studies. In the OlympiAD study, olaparib significantly prolonged progression-free survival in BRCA mutation-positive and HER2-negative patients with inoperable or recurrent breast cancer who had received prior chemotherapy [2]. In the OlympiA study, olaparib predominantly prolonged invasive disease-free survival in BRCA mutation-positive and HER2-negative breast cancer patients with a high risk of recurrence [3]. Olaparib also showed clinical efficacy in ovarian cancer, prostate cancer, and pancreatic cancer [4-6]. In these studies, frequent adverse events were nausea, fatigue or asthenia, anemia, vomiting, and diarrhea [4-6].

Regarding skin rash, the OlympiAD study reported only a 0.5% incidence of palmar-plantar erythrodysesthesia, while no cutaneous adverse events were identified in the OlympiA study [2, 3]. The OlympiAD study also reported a 0.5% incidence of olaparib-induced EN [2], although details were not provided. Excluding those two studies, no skin disorders, including EN, were reported as adverse events [4-6]. EN is considered to occur only rarely as an adverse event associated with olaparib treatment.

EN is a form of septal panniculitis and is thought to be a hypersensitivity reaction to various antigenic stimuli resulting from the formation and deposition of immune complexes in the veins of the subcutaneous adipose septa. Early lesions are characterized by neutrophilic inflammatory infarcts, and activated neutrophils can cause oxidative tissue damage and inflammation through the production of reactive oxygen intermediates. The clinical course is characterized by the acute onset of tender, erythematous nodules or plaques with a diameter of 1-6 cm. Lesions are bilateral and symmetrical and are typically located on the anterior tibiae, although they may also occur on the ankle, thigh, and forearm. The lesions heal within one to six weeks, with the initial bright red discoloration changing to yellowish-brown or greenish-blue bruising. Common causes of EN include drug reactions, strep throat, tuberculosis, sarcoidosis, Behcet’s disease, pregnancy, and inflammatory bowel disease [7]. The incidence of drug-related events is reported to be 2.9-5.0% [10, 11]. In terms of drug types, sulphonamides, bromides, and oral contraceptives are most commonly implicated [7]. One report suggested that the diagnosis should be made based on characteristic symptoms, but skin biopsies should be performed if possible [12]. Non-steroidal anti-inflammatory drugs [13] and sodium iodide [14] are effective for the treatment of EN, and oral steroids should be considered in severe cases [7]. Elastic bandaging and elevation of the lower limbs are also effective in reducing edema and pain [15]. The symptoms of this patient were erythema of the bilateral lower legs, pain, and gait disturbance due to EN, with no other symptoms. Therefore, strep throat, tuberculosis, sarcoidosis, Behcet's disease, and inflammatory bowel disease were considered unlikely causes of EN. Since this patient was menopausal, pregnancy was not considered a possibility. Therefore, drug-related was considered the most likely cause of EN, and the suspected drug was olaparib, which was started four days before onset. It was reported that the relapse of malignancy might induce EN, although the patient’s disease status was relatively stable in this case [16]. Erythema induratum of Bazin is included in the differential diagnosis of EN. It is usually associated with tuberculosis and is characterized by painful subcutaneous erythematous nodules that are usually located on the back of the legs and are prone to necrotic ulceration and scarring. The patient in this case did not have an infectious lung disease such as tuberculosis or necrotic ulceration of the erythematous nodules; thus, a clinical diagnosis of EN was made. There is a case report of EN diagnosed by PET-CT [17], but in this case, PET-CT was not performed at the time of the onset of EN.

There has been only one other case report of olaparib-induced EN [18]. Table 2 shows a comparison of the clinical course in the two cases. In the case reported by Wheelden et al., numerous erythematous nodules with marked tenderness to palpation were observed on both lower limbs three days after starting olaparib. Olaparib was discontinued, paracetamol was administered, and dramatic improvement was observed within 24 hours, with complete resolution of the rash within a week [18]. In the present case, erythema with tenderness appeared on both lower legs after four days of oral olaparib treatment, and the rash improved within seven days of discontinuing olaparib and starting treatment with oral prednisolone.

**Table 2:** Comparison with a previously reported case of olaparib-induced erythema nodosum.

	Time from olaparib administration to onset of erythema nodosum	Symptoms other than skin rash	Rash	Tenderness	Necrotic ulcer	Extent of skin rash	Medical treatment	Time to improvement
Wheelden M et al. [18]	3 days	None	Erythema	+	-	Bilateral lower leg	Acetaminophen	Within 1 week
Our case	4 days	None	Erythema	+	-	Bilateral lower leg	Oral prednisolone 20mg	1 week

As we mentioned above, EN is thought to be a hypersensitivity reaction to various antigenic stimuli [7]. Re-administration of olaparib would likely result in EN. So, we considered that if olaparib is administered again, it should be administered at a reduced dose and with careful follow-up. Also, we considered that if the symptoms of the EN were severe enough to interfere with daily life, the patient should not be readministered in the future.

A PubMed search of the literature using the terms "erythema nodosum" and "breast cancer" identified a case report describing the development of EN in breast cancer patients treated with aromatase inhibitors (AIs) [19,20]. Although the etiology of EN in these patients was not identified, the skin rash disappeared rapidly after AI discontinuation. The clinical course in both cases resembled that in the present case.

## Conclusions

Here we report a rare case of EN that occurred after olaparib administration. The skin rash resolved immediately after olaparib discontinuation. Because EN may develop following therapy with a number of different agents, careful examination is required for patients receiving invasive treatment.
